# Evolving cognition of the JAK-STAT signaling pathway: autoimmune disorders and cancer

**DOI:** 10.1038/s41392-023-01468-7

**Published:** 2023-05-19

**Authors:** Chen Xue, Qinfan Yao, Xinyu Gu, Qingmiao Shi, Xin Yuan, Qingfei Chu, Zhengyi Bao, Juan Lu, Lanjuan Li

**Affiliations:** 1 State Key Laboratory for Diagnosis and Treatment of Infectious Diseases, National Clinical Research Center for Infectious Diseases, National Medical Center for Infectious Diseases, Collaborative Innovation Center for Diagnosis and Treatment of Infectious Diseases, The First Affiliated Hospital, Zhejiang University School of Medicine, Hangzhou, Zhejiang China; 2 Kidney Disease Center, The First Affiliated Hospital, Zhejiang University School of Medicine, Hangzhou, Zhejiang, China

**Keywords:** Tumour immunology, Biomarkers

## Abstract

The Janus kinase (JAK) signal transducer and activator of transcription (JAK-STAT) pathway is an evolutionarily conserved mechanism of transmembrane signal transduction that enables cells to communicate with the exterior environment. Various cytokines, interferons, growth factors, and other specific molecules activate JAK-STAT signaling to drive a series of physiological and pathological processes, including proliferation, metabolism, immune response, inflammation, and malignancy. Dysregulated JAK-STAT signaling and related genetic mutations are strongly associated with immune activation and cancer progression. Insights into the structures and functions of the JAK-STAT pathway have led to the development and approval of diverse drugs for the clinical treatment of diseases. Currently, drugs have been developed to mainly target the JAK-STAT pathway and are commonly divided into three subtypes: cytokine or receptor antibodies, JAK inhibitors, and STAT inhibitors. And novel agents also continue to be developed and tested in preclinical and clinical studies. The effectiveness and safety of each kind of drug also warrant further scientific trials before put into being clinical applications. Here, we review the current understanding of the fundamental composition and function of the JAK-STAT signaling pathway. We also discuss advancements in the understanding of JAK-STAT–related pathogenic mechanisms; targeted JAK-STAT therapies for various diseases, especially immune disorders, and cancers; newly developed JAK inhibitors; and current challenges and directions in the field.

## Introduction

The Janus kinase (JAK) signal transducer and activator of transcription (JAK-STAT) pathway is an evolutionarily conserved signaling pathway that functions in several crucial physiological processes, including hematopoiesis, differentiation, metabolism, and immune modulation.^1–4^ Structurally, the JAK-STAT pathway involves transmembrane receptors, receptor-associated cytosolic tyrosine kinases (i.e. JAKs), and signal transducers and activators of transcription (i.e., STATs).^5^ The JAK protein family contains four members: JAK1, JAK2, JAK3, and TYK2.^6–9^ The STAT family consists of seven proteins: STAT1, STAT2, STAT3, STAT4, STAT5A, STAT5B, and STAT6.^10–12^ The JAK-STAT signaling pathway was first discovered in investigations of interferon-related transcriptional activation. Subsequently, a general outline of the components and pathogenesis of the JAK-STAT signaling pathway was gradually completed over a period of about 20 years.^13–15^ More than 50 types of cytokines, including interferons (IFNs), interleukins (ILs), and growth factors, have been shown to play roles in JAK-STAT signaling to fulfill regulatory functions in cell differentiation, metabolism, survival, homeostasis, and immune response. Once receptors bind to an extracellular ligand, JAKs initiate tyrosine phosphorylation of the receptors and recruit corresponding STATs.^16–18^ The phosphorylated STATs then dimerize and enter the nucleus to regulate specific gene transcription. This process enables the rapid transmission of external signals to the nucleus to regulate biological and pathological processes. Genome-wide association studies for disease exploration have identified more than 200 somatic mutations and single-nucleotide polymorphisms of JAK-STAT pathway genes that are functionally correlated with human diseases, including rheumatoid arthritis (RA), hematological malignancies, and atopic dermatitis (AD).^19–21^ Abnormal activation of JAK-STAT signaling has been identified in diverse immune-mediated conditions and cancers, including melanomas, glioblastomas, and head, neck, lung, pancreatic, breast, rectal, and prostate cancers.^22–27^ Based on the ‘double-edged sword’ function of the JAK-STAT pathway in disease pathogenesis, numerous agents targeting the JAK-STAT pathway have been developed and tested in preclinical and clinical trials.^28–31^ As a result, first-generation JAK inhibitors, such as ruxolitinib, tofacitinib, and baricitinib, have been approved for clinical use.^32–34^ Furthermore, a new generation of selective JAK inhibitors has emerged as a promising option for drug development and has shown success in preclinical trials. Despite this progress, some studies have reported adverse effects of JAK inhibitors, including infection, hematologic events, Wernicke encephalopathy, and even cancer, emphasizing the need for follow-up studies and in-depth investigations on drug thresholds, the mechanisms of adverse events, and drug resistance.^35–37^

In this review, we have expanded and updated the comprehensive research on the components, classical activation, and negative regulation of JAK-STAT signaling based on prior research. We have placed a specific emphasis on the immunomodulatory role of JAK-STAT signaling and the genetic associations between the JAK-STAT signaling pathway and certain diseases. We also discuss in detail the application of JAK inhibitors for the treatment of immune-related and malignant diseases and summarize their efficacy and safety in a series of clinical and preclinical trials.

## JAK, STAT, and the JAK-STAT pathway

The JAK-STAT pathway is an evolutionarily conserved signaling pathway activated by cytokine stimulation that enables extracellular signals to be transmitted across the cell membrane to the nucleus, causing changes in DNA transcription.^38,39^ JAK-STAT signaling regulates a variety of cellular functions, including proliferation, migration, differentiation, and apoptosis.^40,41^ Importantly, JAK-STAT signaling also has an important regulatory role in immune function.^42–44^

## The JAK family

The JAK family of non-receptor tyrosine kinases consists of four proteins: JAK1, JAK2, JAK3, and TYK2.^6,45–47^ Each kinase functions as an intracellular adaptor protein for cytokine signaling.^48–53^ JAK3 is expressed at high levels predominantly in hematopoietic cells, whereas the other members are widely expressed in many tissues.^54,55^ The JAK proteins are composed of the FERM (the complex of four point one, ezrin, radixin, and moesin), Src homology domain (SH2), pseudokinase, and kinase domains.^56–58^ The FERM domain has a clover structure composed of F1, F2, and F3 substructures.^59^ The FERM and SH2 domains are primarily responsible for JAK binding to receptors. The pseudokinase domain regulates the activity of the kinase domain, which is essential for the phosphorylation of receptor tyrosine which leads to further phosphorylation of downstream molecules.^60^ The four domains can be further divided into seven partitions referred to as JH1–7.^45,61,62^ JH1 and JH2 are located at the C-terminal end of the protein, and JH3–7 is located at the N-terminal end.^63–67^ JH1 encodes a kinase that phosphorylates an important component of the kinase domain of the substrate.^68–70^ The main function of JH2, also known as the pseudokinase domain without kinase activity, is to enhance the kinase function of JH1. JH3 and JH4 maintain the stability of the kinase structure.^71–76^ JH5, JH6, and JH7 are responsible for the attachment of JAK to corresponding receptors. Cytokines such as interferons, interleukins, and growth factors and their receptors are the main activators of JAK.^77^ The receptor-ligand complex activates receptor-bound JAK, which catalyzes the phosphorylation of a receptor tyrosine. The four known members of the JAK family each interact with specific cytokine receptors and recruit corresponding STATs to exert diverse biological functions.^78–80^ JAK1, JAK3, and TYK2 are responsible for immune system development and immune regulation, whereas JAK2 mainly participates in hematopoiesis.^81–90^

## The STAT family

STAT proteins are signaling molecules downstream of JAK. The STAT family members are STAT1, STAT2, STAT3, STAT4, STAT5A, STAT5B, and STAT6.^91–94^ STATs consist of an N-terminal domain and coil, a helix domain, a DNA-binding domain, a connection domain, an SH2 domain, and a transcription-activation domain.^95–100^ The N-terminal domain and coil promote the formation of STAT dimers. The helix domain regulates the processes of nuclear import and export.^101–104^ The DNA-binding domain enables STATs to bind DNA as transcription factors.^105^ The SH2 domain recognizes phosphorylated tyrosine on specific cytokine receptors.^106^ After the receptor tyrosine is phosphorylated, cytosolic STATs are recruited to the activated receptor, and a STAT tyrosine is phosphorylated, leading to the formation of STAT dimers.^107–109^ STAT dimers then enter the nucleus as a component of transcription factor complexes to promote the transcription of specific target genes.^110,111^ STATs are then dephosphorylated in the nucleus and returned to the cytoplasm. Within the STAT family, STAT3 has been acknowledged to play a central role in signal transmission from the plasma membrane to the nucleus, making it a promising target for drug development.^112–114^

## Negative regulation of JAK-STAT signaling

**Fig. 1:**
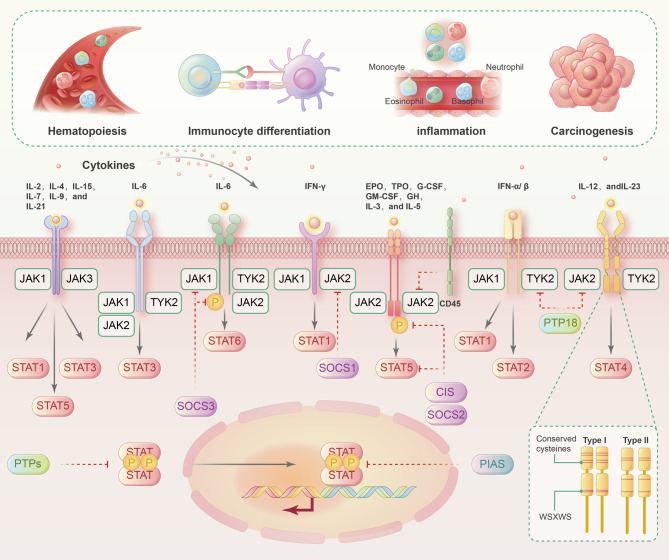
Canonical activation and negative regulation of JAK-STAT signaling pathways. Canonical activation of the JAK-STAT signaling pathway: cytokines bind to their corresponding receptors, which undergo a conformational change and recruit related JAKs. The JAKs undergo phosphorylation, which leads to tyrosine phosphorylation of the receptors to create docking sites for STATs. STATs are phosphorylated, dissociate from the receptor, and enter the nucleus as homodimers or heterodimers to bind specific DNA sites and regulate cytokine-related gene transcription. Negative regulation of the JAK-STAT signaling pathway: the CIS/SOCS (cytokine signaling inhibitor), PIAS (protein inhibitor of activated STAT), and PTP (protein tyrosine phosphatase) families are participants in the negative regulation of the JAK-STAT signaling pathway. The CIS/SOCS family negatively regulates the JAK-STAT pathway through direct binding of JAKs or JAK receptors to inhibit JAK kinase activity, binding of receptor tyrosine kinase sites to intercept STAT-receptor binding, or formation of enzyme complexes to degrade JAKs or STATs. The PIAS family mainly interacts with STAT dimers to inhibit STAT binding to DNA, thereby blocking JAK-STAT signal transduction. The PTP family negatively regulates the JAK/STAT pathway mainly by dephosphorylating activated receptors, JAKs, and STATs. Solid lines indicate the activation process. The dotted lines represent negative regulation

Many regulators inhibit the activation of the JAK-STAT signaling pathway. There are three main types of JAK-STAT regulators: suppressors of cytokine signaling (SOCSs), protein inhibitors of activated STATs (PIASs), and protein tyrosine phosphatases.^115–119^ The SOCS family are the major signaling molecules that attenuate the JAK-STAT pathway and include CIS, SOCS1, SOCS2, SOCS3, SOCS4, SOCS5, SOCS6, and SOCS7.^120–122^ Structurally, all the SOCSs contain an SH2 domain and a SOCS cassette. SOCSs can be induced by cytokines such as IL-2, IL-3, and IFN-γ. The entry of activated STATs into the nucleus enhances the transcription of SOCSs, which exert negative regulatory effects on JAK-STAT signaling by blocking STAT-receptor binding, inactivating JAKs via the N-terminal kinase inhibitory structure, or binding and ubiquitinating JAKs or STATs for proteasomal degradation.^123,124^ A negative feedback loop is driven by the positive effect of activated STATs on the transcription of SOCSs.^125^ The PIAS family includes PIAS1, PIAS3, PIASx, and PIASy.^126–128^ PIASs can interact with STAT to prevent STAT dimerization or prevent STAT dimers from binding to DNA. As phosphatases, protein tyrosine phosphatases can interact with receptors to dephosphorylate JAK. Protein tyrosine phosphatases can also directly dephosphorylate STAT dimers to inhibit JAK-STAT signaling (Fig. 1).^129,130^

## The JAK-STAT pathway, immunoregulation, and lineage plasticity

**Fig. 2:**
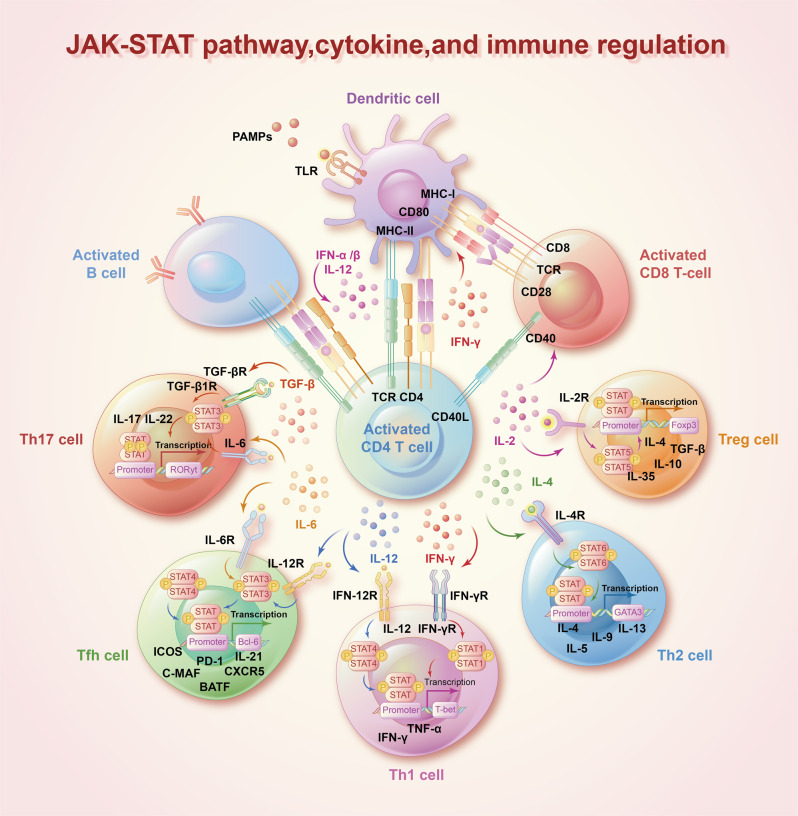
The JAK-STAT signaling pathway and immune. Interactions between a large number of cytokines and the JAK-STAT pathway influence immune cell differentiation and development and exert immunoregulatory effects. IFN and IL-12 are crucial for Th1 cell differentiation and drive T-bet gene expression through STAT1 and STAT4, respectively. IL-4 upregulates GATA3 genes via STAT6 to activate Th2 cell differentiation. IL-6 and TGF play an essential role in Th17 cell differentiation via STAT3 to trigger RORt gene expression. IL-6 and IL-12 influence T follicular helper cell differentiation via STAT3 to increase Bcl-6 transcription. IL-2 induces Treg cell differentiation via the direct combination of STAT5A/B with the Foxp3 gene

**Fig. 3:**
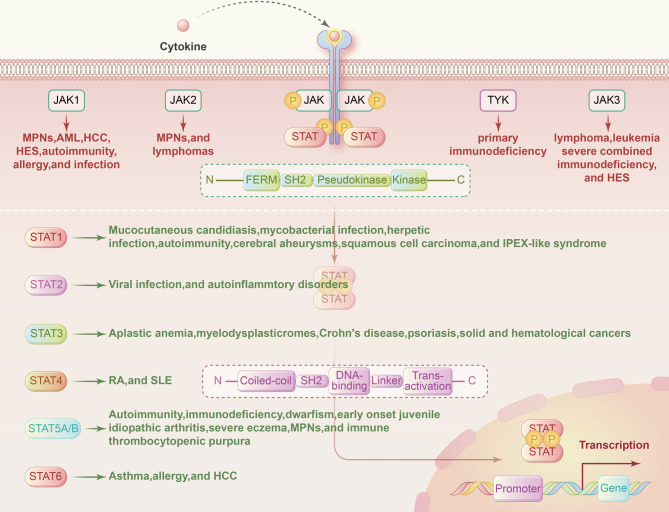
Mutations of the JAK-STAT pathway in human disease. Genetic mutations and polymorphisms of genes in the JAK-STAT pathway are widely involved in the pathogenesis of human diseases. The most frequent site of disease-causing mutations is within the SH2 domain in both JAKs and STATs

Cytokines are crucial for humoral and cellular immune responses.^44,131,132^ A wide range of cytokines associated with autoimmune diseases, including IFNs, ILs, and colony-stimulating factors, exert pleiotropic effects primarily through a combination of type I and type II cytokine receptors.^133–135^ The interaction of type I and type II cytokine receptors can activate JAKs and subsequently recruit STATs to transduce signals from cytokines (Table 1).^1,136,137^ Numerous studies have shown that cytokine-induced activation of the JAK-STAT pathway plays instrumental roles in the differentiation and development of immune cells and the homeostasis of the immune system (Fig. 2).^138–140^ Under stimulation by different cytokines, the JAK-STAT signaling pathway performs complex functions in immune regulatory events, these functions include not only cancer cell recognition, triggered mainly by IFNs and STAT1 and STAT2 signaling, but also immune escape triggered mainly by IL-6-STAT3 signaling.^23^ JAK-STAT signaling has been widely shown to drive antitumor immune surveillance in response to cytokines (like IL-2, IL-15, and IFNs) by inducing the activation, cytotoxicity, and function of natural killer (NK) cells.^141–144^ On the other hand, the JAK-STAT pathway has been implicated in the pathogenesis of numerous autoimmune diseases such as RA, inflammatory bowel disease, and AD.^145,146^ For example, IL-6 induced the phosphorylation of STAT3 and upregulated the differentiation of Th17 cells, contributing to RA development.^147^ Ma, C. S. et al. also showed that STAT3 deficiency resulted in the production of incomplete T follicular helper cells and reduction of B-cell helper activity.^148^ In animal models of spondyloarthritis (SpA), TYK2 was shown to be an essential mediator of IL-22–induced STAT3 phosphorylation to enhance type 3 immunity and accelerate SpA progression.^149^ Furthermore, multiple genome-wide association analyzes have clearly demonstrated that polymorphisms and mutations in the JAK-STAT pathway are associated with autoimmune diseases and immune-mediated cancers (Fig. 3).^19,150,151^ Casaca, V. I. et al. reported that the rs324011 polymorphism in STAT6 resulted in early immune dysregulation with depressed Treg function and increased Th1 response at birth.^152^ Fabre, A. et al. reported links between STAT3 gain-of-function mutations and early-onset polyautoimmunity.^153^ Mutated STAT3 was also reported to participate in the pathogenesis of immune-mediated aplastic anemia by altering the T-cell phenotype and giving cytotoxic properties to CD8+ T cells.^154^ Additionally, JAK mutations were revealed to block IFN-γ signal transmission, contributing to immune evasion and insensitivity to anti-PD-1/PD-L1 immunotherapy.^155^ Recent clinical trials have shown that targeting JAKs and preventing their phosphorylation suppresses abnormal immune and inflammatory responses caused by cytokines, providing reasonable and solid evidence to support the use of JAK inhibitors as therapies to treat autoimmune diseases and cancers.

**Table 1:** The associated cytokines and diseases of JAKs and STATs

JAKs and STATs	Associated cytokine	Associated diseases	Ref.
JAK1	IL-2, IL-4, IL-6, IL-7, IL-9, IL-10, IL-11, IL-15, IL-19, IL-20, IL-21, IL-22, IL-24, IL-28, IL-29, CNTF, OSM, LIF, CT-1, IFNα/β, and IFNγ	MPNs, AML, HCC, HES, autoimmunity, allergy, and infection	^63–67^
JAK2	IL-3, IL-5, IL-6, IL-10, IL-11, IL-12, IL-13, IL-19, IL-20, IL-22, IL-23, IL-27, GH, EPO, TPO, PRL, Leptin, G-CSF, and GM-CSF	MPNs, and lymphomas	^71–75^
JAK3	IL-2, IL-4, IL-7, IL-9, IL-15, and IL-21	lymphoma, leukemia, severe combined immunodeficiency, and HES	^48–53^
Tyk2	IL-6, IL-10, IL-11, IL-12, Il-13, IL-23, IL-27, IL-19, IL-20, IL-22, IL-28, IL-29, IFN-α/β, and IFNγ	primary immunodeficiency	^9,64,85–90^
STAT1	IL-2, IL-6, IL-10, IL-11, IL-22, IL-27, IL-28, IL-29, PDGF, EGF, HGF, TNF, TPO, angiotensin II, IFN-α/β, and IFNγ	mucocutaneous candidiasis, mycobacterial infection, herpetic infection, autoimmunity, cerebral aneurysms, squamous cell carcinoma, and IPEX-like syndrome	^95–100^
STAT2	IL-28, IL-29, and IFNα/β	viral infection, and autoinflammatory disorders	^42,76,110,111^
STAT3	IL-2, IL-3, IL-5, IL-6, IL-7, IL-9, IL-10, IL-11, IL-15, IL-19, IL-20, IL-21, IL-22, IL-23, IL-24, IL-26, IL-27, IL-28, IL-29, IL-31, LIF, CNTF, CT-1, OSM, CLCF1, GH, TPO, G-CSF, GM-CSF, Leptin, and IFNα/β	aplastic anemia, myelodysplastic syndromes, Crohn’s disease, psoriasis, solid and hematological cancers	^321,371–374^
STAT4	IL-12, IL-23, IL-27, and IFNα/β	RA and SLE	^355–358^
STAT5a, STAT5b	IL-2, IL-3, IL-4, IL-5, IL-7, IL-9, IL-10, IL-15, IL-21, IL-22, IL-27, IL-28, IL-29, EGF, EPO, G-CSF, GM-CSF, TPO, GH, PDGF, Prolactin, and Leptin	autoimmunity, immunodeficiency, dwarfism, early-onset juvenile idiopathic arthritis, severe eczema, MPNs, and immune thrombocytopenic purpura	^362–367^
STAT6	IL-3, IL-4, IL-5, and IL-13	asthma, allergy, and HCC	^101–104^

The effects of the JAK-STAT pathway on disease progression are complex. Cellular plasticity enables cells to adopt new phenotypes in response to environmental changes.^156–158^ Cancer cells amplify this plasticity to cause tumor heterogeneity, metastasis, and therapy resistance.^156,159,160^ The histologic transformation of lung cancers harboring epidermal growth factor receptor (EGFR) mutations from adenocarcinoma to aggressive neuroendocrine cancer is a notable example of lineage plasticity in cancer.^161,162^ Numerous lines of evidence suggest that JAK-STAT signaling is tightly connected to lineage plasticity and resistance through stem cell self-renewal modulation and multilineage differentiation.^163–165^ One of the most outstanding examples of this is the contribution of activated JAK-STAT signaling to the lineage transition of prostate cancer from adenocarcinoma to neuroendocrine cancer.^31,166^ In liver cancer, JAK-STAT3 is involved in the RAS-induced, malignancy-associated trans-differentiation of hepatocytes into intrahepatic cholangiocarcinoma cells.^167^ Moreover, activation of the IL-6-JAK-STAT pathway by WNT5A is known to facilitate epithelial-mesenchymal transition (EMT) in keloid scarring.^160^ A more comprehensive understanding of the effects of the JAK-STAT signaling pathway on lineage plasticity will provide a plausible molecular basis for the development of new therapies for malignant diseases.^168^

## The JAK-STAT pathway and diseases

There is emerging evidence that JAK-STAT activation can play dual roles in diseases. Hyperactivation of the JAK-STAT pathway has been implicated in the poor outcomes of many diseases including melanomas, glioblastomas, and head, neck, lung, pancreatic, breast, rectal, and prostate cancers.^23,125,169^ Conversely, favorable regulatory roles of the JAK-STAT pathway have been demonstrated in head and neck squamous cell carcinomas and prostate and colorectal cancers. In the next sections, we have highlighted the critical roles of the JAK-STAT pathway in some common diseases, including rheumatoid arthritis, myeloproliferative neoplasms, kidney diseases, and prostate, breast, and lung cancers.

### Rheumatoid arthritis

**Fig. 4:**
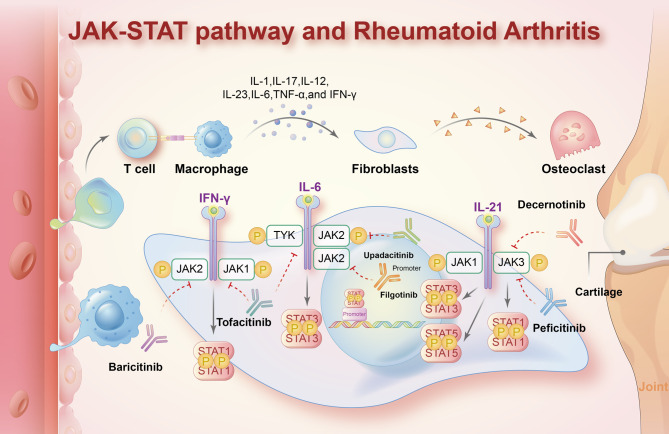
Roles of the JAK-STAT pathway in the pathogenesis of rheumatoid arthritis and recently approved JAK inhibitors. Abnormal JAK-STAT signaling induced by multiple cytokines is regarded as the essential pathogenesis of RA. The interaction of many cytokines (including IL-1, IL-17, IL-12, IL-23, IL-6, TNF-α, and IFN-γ) and the JAK-STAT pathway mediates inflammatory responses in the synovium and causes joint destruction. There are several JAK inhibitors approved for RA treatment, including tofacitinib, baricitinib, filgotinib, upadacitinib, peficitinib, and decernotinib. The dotted lines indicate negative regulation

RA is a chronic inflammatory joint disease marked by progressive synovitis, leading to irreversible cartilage and bone erosion, eventual joint destruction, and functional disability.^170–172^ A central feature of RA pathogenesis is the production of inflammatory cytokines such as TNF, IL-1, and IL-6 by synovial cells.^173–175^ Several studies have reported that multiple proinflammatory cytokines drive RA progression by activating JAK-STAT signaling (Fig. 4).^176–179^ Fibroblasts, the key drivers of inflammation in the RA-afflicted synovium, were confirmed to produce IL-6 in the presence of STAT4 activation to trigger continuous joint destruction.^180^ Mori T. et al. pointed out that the IL-6–STAT3 cytokine loop is activated by inflammatory cytokines that are highly expressed in RA, causing chronic and persistent inflammation and joint destruction.^181^ Fibroblast-like synoviocytes from patients with active RA display elevated STAT1 expression and activity.^182,183^ Increased STAT3 activity in synovial CD4^+^ T cells in RA results in increased numbers of Th17 cells and reduced numbers of Treg cells and is closely related to the severity of synovitis. JAK3, STAT4, and STAT6 are also highly expressed in CD1a^+^ dendritic cells of patients with RA and may help to identify RA at the synovium level.^184–188^ Mouse models of proteoglycan-induced arthritis revealed that IL-4 controls inflammatory responses in RA by inhibiting IL-12-STAT4 signaling. Additionally, a gene ontology and pathway analysis revealed increased expression of type I IFN-inducible genes in the peripheral blood of patients with RA.^189^ Higgs, B. W. et al. demonstrated that an overactive type I IFN pathway is involved in the disease activity of RA.^190^ Another study showed that miR-17 suppressed the secretion of proinflammatory cytokines such as IL-6 and IL-1β and bound to STAT3 and JAK1 to play an anti-inflammatory and anti-erosive role in RA development. Wang, J. et al. reported that long intergenic non-protein-encoding long-chain RNA p53-induced transcript (LncRNA LINC-PINT) suppressed TNF-α–induced synovial fibroblasts in RA by upregulating SOCS1 levels.^191^

JAK inhibitors were recently introduced as a novel type of disease-modifying antirheumatic drugs (DMARDs), which reduce synovitis and systemic inflammation and improve function in RA (Table 2). The European Society of Rheumatology (EULAR) in 2019 equated the application of JAK inhibitors with that of biological DMARDs (bDMARDs) in circumstances where conventional synthetic DMARDs (csDMARDs) are ineffective.^192–194^ A large observational study of the efficacy of commonly used second-line RA drugs confirmed that JAK inhibitors were more effective than methotrexate.^195–198^ Chen, C. et al. recently proposed that the JAK3 inhibitor Z583 irreversibly combines with cysteine residue 909 (Cys909) of JAK3 to prevent activation of JAK-STAT signaling, exerting powerful suppressive effects on RA progression.^199^ Given the expression and influence of JAK-STAT signaling in RA, JAK inhibitors are regarded as appropriate agents for RA management, although further large-scale studies are required to define more specific clinical applications of agents targeting JAK-STAT signaling in RA.^200–205^

**Table 2:** Four licensed JAKis for RA treatment

JAKis	Main trials	Chemical structure	*T*_1/2_	Excretion	Clinically used doses	ref.
Baricitinib	Phase III, Genovese et al. (RA-BEACON); Phase III, Dougados et al. (RA-BUILD); Phase III, Fleischmann et al. (RA-BEGIN); and Phase III, Taylor et al. (RA-BEAM)		9–13 h	Urine75%, and Stool20%	2 mg/4 mg	^475–478^
Tofacitinib	ORAL program: Phase III, Fleischmann et al. (ORAL solo); Phase III, Burmester et al. (ORAL step); Phase III, Vollenhoven et al. (ORAL-standard); Phase III, Kremer et al. (ORAL sync); Phase III, van der Heijde et al. (ORAL scan); and Phase III, Lee et al. (ORAL start)		3 h	Urine51%, and Stool13%	5 mg/10 mg	^200–205^
Filgotinib	Phase III, Genovese et al,(FINCH 2 trial), and Extension Study of Phase II, Kavanaugh et al. (DARWIN 3)		6 h	Urine87%, and Stool15%	100 mg/200 mg	^177,178,196,197^
Upadacitinib	Phase III, Cohenet et al. (SELECT program)		8–14 h	Urine24%, and Stool38%	15 mg/30 mg	^261,262,265,266^

### Myeloproliferative neoplasms

Within hematology, JAK-STAT signaling has been explored in the context of myeloproliferative neoplasms (MPNs).^206–209^ MPNs are clonal hematopoietic conditions characterized by excessive proliferation of myeloid lineage cells, which contributes to abnormal numbers and morphology of peripheral blood cells and a high-risk of acute myeloid leukemia development.^210–213^ High-resolution genome-wide genotyping has identified acquired somatic mutations that are associated with increased JAK-STAT activity in various types of MPNs.^214–216^ Numerous studies reported that JAK2 mutations are frequent in MPNs and exert disruptive effects on the regulation of diverse biological processes.^217–222^ Rampal R et al. employed an integrated genomic analysis and found that the distinctive upregulation of JAK-STAT target genes helped distinguish different subtypes of MPNs, and the degree of valine-to-phenylalanine substitution at amino acid position 617 of JAK2 (JAK2^V617F^) influenced the disease severity.^223^ JAK2^V617F^ results in constitutive tyrosine phosphorylation activity and is the most prevalent gain-of-function alteration in the majority of MPNs.^61,224–226^ Experiments with Pf4-Cre transgenic mice indicated that activated JAK-STAT signaling in megakaryocytes with the JAK2^V617F^ mutation promoted the induction and maintenance of myeloproliferation in MPN through the production of proinflammatory cytokines and chemokines.^227^ Based on the association between JAK2^V617F^ and MPN, the World Health Organization Classification and the International Consensus Classification both proposed a novel definition of “JAK2 mutation-prevalent MPNs”, which mainly consist of polycythemia vera, essential thrombocythemia, primary myelofibrosis, and unclassifiable MPN.^219,228^ Genomic analysis revealed frequent JAK2^V617F^ mutations in over 90% of polycythemia vera cases and about 50% of essential thrombocythemia and primary myelofibrosis cases.

Calreticulin (CALR), a chaperone protein that localizes in the endoplasmic reticulum and participates in protein folding, is the second most frequently mutated protein in MPNs after JAK2. It is well established that CALR mutation is mutually exclusive with other MPN driver mutations, including JAK2^V617F^ and the thrombopoietin receptor (MPL) mutation MPL^W515L^.^229–231^ Whole-exome sequencing identified CALR mutations in ~70% to 80% of JAK2^V617F^-negative ETs and primary myelofibrosis.^232,233^ In vivo research demonstrated that CALR mutation alone was strong enough to initiate an MPN phenotype and increased JAK-STAT signaling activity. The cellular model revealed that CALR mutants specifically activated MPL to drive the pathogenesis of MPN. Elf, S. et al. further demonstrated that the malignant transformation driven by mutant CALR in MPN required interaction with MPL.^234,235^

Thrombopoietin and its receptor MPL are primary regulators of megakaryocyte growth and differentiation.^236–238^ The pathogenetic mutation MPL^W515L^ induces abnormal activation of JAK-STAT signaling and participates in MPN development.^231,239^ MPL^W515L^ promotes hematopoietic cell proliferation and cellular responses to thrombopoietin. Lnk, the negative regulator of thrombopoietin, can reverse the growth-stimulating signals in cells carrying MPL^W515L^ and cause malignant cells to die. Moreover, a study of a cohort of 1182 patients with MPN and the exploration of bone marrow-derived DNA from different disease courses consistently showed that MPL^W515L^ and JAK2^V617F^ occurred concurrently in MPNs, suggesting that the functions of these driver mutations may be relatively complementary in the course of MPN pathogenesis.^240,241^

### Atopic dermatitis

Atopic dermatitis (AD) is one of the most common chronic immune-mediated skin conditions. Patients with AD suffer recurrent symptoms and impaired quality of life.^242–244^ Existing therapies for AD relieve the symptoms but cannot delay disease progression, and all of these therapies have limitations in clinical applications.^245,246^ The pathogenesis of AD has a multifactorial origin and is considered to predominantly result from T helper 2 (Th2) cell-mediated inflammation and the gradual upregulation of Th1, Th17, and Th22 cell-mediated inflammation at the later stages of disease progression.^247^ A variety of mediators, including IL-4, IL-13, IL-31, and TSLP, bind specific transmembrane receptors and stimulate the JAK-STAT pathway, thereby initiating intracellular signaling and exerting proinflammatory effects in AD.^248,249^ In-depth explorations of AD pathogenesis have suggested that the JAK-STAT pathway is involved in Th2 immune polarization, eosinophil activation, and skin barrier destruction during AD progression.^250^

A greater understanding of the roles of the JAK-STAT pathway in AD has enabled the application of JAK inhibitors as a new approach to AD treatment.^251–253^ Baricitinib, an oral selective inhibitor of JAK1 and JAK2, has been approved for the treatment of moderate-to-severe AD.^254^ The results of two phase 3 trials (BREEZE-AD1 and BREEZE-AD2) have shown that baricitinib significantly relieves unbearable pruritus and skin lesions in patients with moderate-to-severe AD.^255^ Topical ruxolitinib has also shown anti-inflammatory and antipruritic effects in experimental models and phase 3 clinical trials (TRuE-AD1 and TRuE-AD2).^256–258^ Given the close connection between JAK1 and the pathogenesis of AD, selective JAK1 inhibitors are promising therapeutic candidates for AD. Upadacitinib, was a highly specific JAK1 inhibitor previously explored in RA and further approved by the FDA in 2019 for the treatment of moderate-to-severe refractory AD in patients aged ≥12 years.^259–268^ Mechanistically, upadacitinib relieves chronic dermatitis by potently attenuating the cytokines related to JAK1 inflammatory signaling (including IL-6 and IFNs).^269–272^ The minimal effects of upadacitinib on JAK2 and JAK3 result in a few side effects, especially in the hemopoietic system.^273–278^ Several phase 3 trials (Measure Up 1, Measure Up 2, and AD Up) have shown that upadacitinib as a monotherapy or in combination with topical corticosteroids has favorable efficacy and tolerability in patients with moderate-to-severe AD.^259,279^ In 2022, abrocitinib, a selective JAK1 inhibitor, was also approved for the treatment of moderate-to-severe AD.^280–283^ Phase 3 studies including JADE MONO-1, MONO-2, and TEEN have shown that 200 or 100 mg abrocitinib was more effective than placebo or dupilumab in terms of the early alleviation of itch and reduction of the eczema area as well as the severity index (EASI-75) and Investigator’s Global Assessment (IGA) scores.^284–288^ A phase 2 study (NCT02201524) also suggested that abrocitinib resulted in favorable symptom improvement in patients with moderate-to-severe psoriasis.^289^

### Hepatocellular carcinoma

Several studies have indicated that the JAK-STAT signaling pathway is widely involved in multiple types of solid cancers. Hepatocellular carcinoma (HCC) is a lethal and heterogeneous tumor with consistently high incidence and mortality rates, contributing to more than 700,000 deaths annually.^290–292^ Chronic hepatitis B virus (HBV) infection occurs in more than 400 million people across the globe and is the most frequent cause of HCC. Despite improvements in the management of HCC, the clinical prognosis of HCC remains unfavorable, with a 5-year survival rate of 10%. The process of hepatic carcinogenesis is thought to involve a series of genetic alterations and abnormal changes in signaling pathways.^293–297^ The JAK-STAT signaling pathway has been extensively explored for potential roles in HCC pathogenetic progress. The use of interferon-alfa (IFN-α) to drive host antiviral responses is the current first-line treatment for chronic hepatitis B and has been verified to slow the progression of liver fibrosis and even the occurrence of HCC. As a crucial immune-related cytokine, IFN-α activates JAK-STAT signaling to induce various IFN-stimulated genes with antiviral and immunomodulatory functions.^298^ Han M et al. found that the expression and activation of IFN-stimulated genes including STAT1, myxovirus resistance (Mx), and SOCS3 in the peripheral blood of patients with chronic hepatitis B were closely correlated with IFN-induced antiviral outcomes.^299,300^ SOCS3 overexpression following HBV infection caused the inactivation of IFN signaling and was correlated with the severity of liver inflammation.^301,302^ In an HBV-producing HepG2 cell line, Hepatitis B Virus X protein weakened IFN-α signaling by inactivating STAT3 to increase SOCS3 and protein phosphatase 2 A expression. Gao D et al. also observed downregulation of SOCS3 by miR-122 increased the anti-HBV effectiveness of IFN.^303^ Numerous studies reported that SOCS3 is downregulated in HCC tissues and is negatively implicated in HCC malignant transformation.^304,305^ SOCS3 methylation was discovered to be more frequent in HBV-positive HCC than in normal liver tissues and was associated with poor HCC outcomes. Eyes absent homolog 2, which transcriptionally upregulates SOCS3 expression to block JAK-STAT signaling, was identified as a potential tumor suppressor in HCC and found to inhibit HCC progression.^306^ Conversely, CircSOD2 suppressed SOCS3 expression and triggered JAK2–STAT3 signaling to drive HCC progression.^307^ Furthermore, as a member of the B7 family of immune checkpoint proteins, HHLA2 was demonstrated to activate the JAK-STAT pathway and accelerate HCC progression by binding to TMIGD2.^308^

### Prostate cancer

Androgen receptor (AR)-targeted therapies such as enzalutamide have achieved considerable clinical success in patients with prostate cancer, mainly by attacking the glandular luminal cells. Unfortunately, advanced prostate cancer usually undergoes lineage conversion into squamous cell carcinoma or neuroendocrine cancer, resulting in drug resistance. Several studies have found associations between excessive JAK-STAT signaling activation in prostate cancer and the regulation of lineage plasticity, resistance to AR-targeted therapies, and poor clinical outcomes.^309,310^ JAK1, JAK2, STAT1, and STAT3 are essential drivers of lineage plasticity in cancer cells. In particular, constitutively activated STAT3 promotes lineage plasticity and associated cell migration and invasion.^311,312^ In mouse organoids, genetically engineered mouse models (GEMMs), and human cell lines, combined with fibroblast growth factor receptor (FGFR) signaling, the JAK-STAT pathway was shown to trigger luminal-basal phenotype conversion and subsequent castration-resistant prostate cancer (CRPC) formation.^31^ The pharmacological inhibition of JAK-STAT signaling in vitro and in vivo led to the re-sensitization of resistant cancer cells to enzalutamide and effectively reversed the undesirable phenotypic plasticity.^166^ These results provide vital theoretical support for new approaches to overcome drug resistance in CRPC and shed light on the association between lineage plasticity and carcinogenesis.

### Breast cancer

Breast cancer is the most prevalent cancer in women worldwide. There are three general categories of breast cancer: hormone receptor-positive (HR+), human epidermal growth factor receptor-positive (HER2+), and triple-negative (TN).^313–315^ Distant metastasis and therapy resistance are the root cause of the poor prognosis of the majority of patients with breast cancer.^316,317^ Accumulating evidence suggests that all the STAT family members are closely associated with breast cancer, having either pro-tumorigenic or antitumorigenic characteristics.^318^ Therefore, breast cancer provides an example of the double-edged sword role of JAK-STAT in carcinogenesis.

As an essential regulator of cell transformation and apoptosis during breast development, STAT3 has been extensively explored in the context of breast cancer.^319,320^ Several studies have shown that STAT3 plays a central role in the proliferation, invasion, metastasis, and immune escape of breast cancer cells.^321,322^ Moreover, STAT3 helps maintain the phenotype and function of breast cancer stem cells (BCSCs), which display pluripotent properties, resistance to chemotherapy, and high capacity for self-renewal.^323–326^ The classical IL-6-JAK-STAT3 pathway upregulates genes related to breast cancer progression, induces resistance to aromatase inhibitor (AI) chemotherapy, and correlates with poor survival in HR+ breast cancer.^327–330^ In patients with HER2+ breast cancer, alone or in combination with trastuzumab, the selective JAK1/2 inhibitor ruxolitinib weakens cancer cell viability and improves the clinical outcomes by inhibiting the IL-6–JAK2–STAT3–calprotectin axis.^331^

Despite the pro-tumorigenic effects of JAK-STAT signaling, some preclinical studies have shown that JAK inhibitors impair the antitumor immunity of NK cells and increase the metastatic burden in breast cancer.^332^ Furthermore, ruxolitinib, a JAK1 and JAK2 inhibitor, can induce the generation of proinflammatory mediators by macrophages, resulting in the establishment of a pro-tumorigenic microenvironment and drug resistance in breast cancer.^333^ The diverse effects of the JAK-STAT pathway in breast cancer highlight the importance of JAK-STAT signaling and offer insights into the development of effective therapeutic strategies.

### Kidney diseases

With the notable exceptions of RA, hematological malignancies, and solid cancers, it’s also reported by the transcriptome analysis that increased STAT1 and STAT3 expression in kidney glomerular and tubulointerstitial sections were associated with disease progression in focal segmental glomerulosclerosis.^334^ Pang et al. confirmed that STAT3 activation mediated renal fibrosis in unilateral ureteral obstruction models, and the application of the novel STAT3 inhibitor S3I-201 suppressed renal interstitial fibroblast activation and fibrosis.^335^ By contrast, Koike, K. et al. observed that activated JAK-STAT3 signaling in unilateral ureteral obstruction models induced matrix metalloproteinase-2 expression in proximal tubular cells to relieve renal fibrosis and promote tissue repair.^336^ The conflicting findings regarding the functions of JAK-STAT signaling in renal tissue repair and fibrosis may result from differences in animal models and the interventions applied to regulate the JAK-STAT pathway.

STAT3 activation induced by hyperuricemia in tubular and interstitial cells was accompanied by kidney fibrosis and dysfunction, and the STAT3 inhibitor S3I-201 was confirmed to suppress the JAK-STAT pathway to achieve anti-fibrotic effects. In an exploration of kidney tubulointerstitial fibrosis, miR-150–based RNA interference was confirmed to reverse activation of the SOCS1-JAK-STAT pathway and alleviate tubulointerstitial fibrosis.^337^ In addition, overactive STAT1 and STAT3 expression and activated JAK-STAT signaling were demonstrated to accompany progressive kidney inflammation and proteinuria in IgA nephropathy.^338^

Activation of JAK-STAT signaling was also found to play an essential pathogenic role in renal damage caused by diabetes mellitus through the stimulation of excessive glomerular mesangial cell growth.^339–341^ Increased activity of the JAK-STAT pathway in mice with diabetic nephropathy prevented podocyte autophagy and enhanced disease progression.^342^ A remarkable increase in JAK2 level was also integrally linked to a series of deteriorating clinical symptoms of diabetic nephropathy, including albuminuria, glomerulosclerosis, and reduced numbers of podocytes.^343^ In STZ-induced diabetic rat models, hyperglycemia triggered the JAK-STAT pathway in glomerular mesangial cells through angiotensin II, and treatment with angiotensin II blockade reversed pathological changes to improve kidney function.

Autosomal dominant polycystic kidney disease (ADPKD), the most common congenital kidney disorder, is commonly accompanied by abnormal activity of JAK-STAT pathways.^344,345^ Forced STAT5 expression in ADPKD causes aberrant proliferation by transcriptionally upregulating cyclin D1 in a growth hormone-dependent manner. Pkd1^nl/nl^ murine models revealed heterotopic JAK2 expression in cyst-lining cells and interstitium and validated that JAK2 inhibition could postpone cystic growth in ADPKD.^346^

Experiments using an MRL/lpr mouse model indicated that renal CD8^+^ tissue-resident memory T cells required JAK/STAT signaling for self-renewal and effector functions, which were related to lupus nephritis activity. The STAT3 level was also dramatically elevated in lupus nephritis and linked to unfavorable clinical parameters.^347^ In non-human primate allograft models based on calcineurin-inhibitor-free regimens, use of the JAK3 inhibitor CP-690,550 in combination with mycophenolate mofetil lowered the incidence of kidney acute rejection and prolonged allograft survival.^348–350^ Additionally, JAK-STAT signaling triggered by IFN-γ was demonstrated to be inhibited by the FGFR pathway in renal cell carcinoma cells, and treatment with lenvatinib, a targeted FGFR receptor tyrosine kinase inhibitor, recovered antitumor immunity and enhanced the efficacy of immune checkpoint inhibitors.^351^

## JAK-STAT pathway and treatment

The JAK-STAT signaling pathway is the essential intracellular route through which diverse extracellular soluble molecules bind membrane receptors and transfer signals to the nucleus. Given its primary roles in a series of cancers and autoimmune diseases, JAK-STAT signaling has emerged as a prominent target for drug development. Drugs that target the JAK-STAT pathway can be classified into three groups according to how they affect the signal transduction process: cytokine or receptor antibodies, STAT inhibitors, and JAK inhibitors.^352–354^

Upstream cytokines and receptors are pivotal for modulating the functions of the JAK-STAT signaling pathway.^355–358^ Drugs that manipulate JAK-STAT-dependent cytokines and receptors (e.g., siltuximab and tocilizumab for the classical blockade of IL-6) inhibit JAK-STAT signal transduction and have therefore been used as therapeutic interventions in many diseases.^359–362^

Most STAT inhibitors function by restricting STAT phosphorylation, inhibiting SH2-mediated dimerization, or inducing STAT degradation.^363–367^ Given the crucial functions of activated STAT3 and STAT5 in signal transduction and pathogenic progression, multiple inhibitors targeting STAT3 and STAT5, including peptides, peptidomimetics, oligonucleotides, siRNAs, small molecules, and metal-based complexes, have shown promising preclinical efficacy.^368–374^ Hundreds of research articles have described the various underlying mechanisms of STATs in disease progression and the efficacies of dozens of STAT inhibitors in preclinical and clinical trials.

STAT3, the most widely studied STAT protein, is frequently mutated and overactivated in diverse cancers and promotes cancer cell proliferation, invasion, metastasis, and immune evasion.^318^ Napabucasin (BBI608), the first direct STAT3 inhibitor to enter phase 3 clinical trials and the first-in-class cancer stemness inhibitor, has been shown to restrain diverse malignant processes in multiple cancers. Moreover, napabucasin was found to decrease the expression of critical stemness-related genes, deplete cancer stem cell (CSC) populations and re-sensitize chemo-resistant cells to cisplatin in cisplatin-resistant NSCLC models.^375^ Napabucasin also possesses anti-acute myeloid leukemia (AML) properties and enhanced the Bcl-2 inhibitor efficacy by inhibiting STAT3 and inducing DNA damage, as revealed in in vitro and in vivo experiments.^376^ In addition, napabucasin eliminated the immunosuppressive functions of both murine and human myeloid-derived suppressor cells (MDSCs) in malignant melanoma and was found to be associated with the patients’ clinical outcomes.^377^ A recent phase 3 trial (NCT01830621) of napabucasin monotherapy in advanced colorectal cancer showed that its potential outcomes in terms of patient prognosis were better in the napabucasin group than in the placebo group.^378^ Moreover, napabucasin has been recently approved by the FDA as an orphan drug for gastric and pancreatic cancer treatment. And OPB-31121, a novel STAT3 inhibitor, induces cell apoptosis and was shown to exert synergism with 5-fluorouracil and cisplatin in both gastric cancer cells and xenograft models.^379^ Additionally, OPB-51602, a selective inhibitor of STAT3 phosphorylation, reduces complex I activity and increases ROS concentrations to maintain high toxicity to cancer cells.^380^ OPB-51602 was observed to improve acquired drug resistance by preventing the reliance of cancer cells on mitochondrial oxidative phosphorylation (OXPHOS).^381,382^ In a first-in-human phase I clinical trial (NCT01184807), OPB-51602 exhibited promising antitumor activity among patients with refractory solid tumors, especially NSCLC.^383^ Moreover, TTI-101 (C188-9), another STAT3 inhibitor, was found to attenuate the activation of the phosphotyrosine (pY) peptide-binding site within the SH2 domain of STAT3, without the influence of mitochondrial function.^384^ Multiple studies conducted in animal models have demonstrated effective therapeutic potential and favorable safety profiles in a series of autoimmune diseases and cancers, including SSc, Crohn’s disease, head and neck cancer, and lung, breast, colorectal, and liver cancers.^385–389^ Most recently, TTI-101 has been granted the ‘fast track’ designation by the FDA for applying relapsed/refractory locally advanced, unresectable, or metastatic hepatocellular carcinoma. An ongoing phase 1 clinical trial of TTI-101 (NCT03195699) has reported that patients with advanced solid tumors tolerated the administration of TTI-101 well.

Moreover, emerging studies have revealed the potential of STAT5 inhibitors. A prior study has demonstrated that STAT5 programs the new generation of GM-CSF-producing T helper (TH) cells (TH-GM), which cause more severe neuroinflammation than TH1 and TH17 cells.^390^ The preliminary result of a STAT5 inhibitor clinflamozyde in a recent COVID-19 clinical trial approved by the FDA observed that clinflamozyde selectively repressed the STAT5-TH-GM pathway to relieve severe cytokine storms and significantly improved clinical outcomes of patients with COVID-19.

Moreover, increasing strategies integrating STAT3 with immune checkpoint inhibitors (e.g., anti-CTLA-4 and PD-1/PD-L1 antibodies), CAR-T-cell therapy (NCT02906371), stimulator of interferon genes (STING) agonists, and even cancer vaccines have shown encouraging synergistic antitumor efficacy in diverse preclinical trails.^319,391–394^

Based on the previous exploration, STAT inhibitors have demonstrated promising value in their antitumor effects. Unfortunately, most STAT inhibitors are still in the preclinical stages of development, and few have been approved for clinical application owing to the lack of intrinsic catalytic activity and selectivity shown by most candidates. A large number of clinical trials targeting STATs as monotherapies and combination therapies are currently underway, offering an attractive drug target for disease treatment.

Despite this, natural products and their derivatives have served as sources of novel antitumor agents based on the JAK-STAT pathway.^395–397^ Over the past several decades, in vitro and in vivo experiments have shown that various natural products have inhibitory effects on the STAT3 signaling pathway and exhibit promising anticancer activities.^398^ For example, the phytochemical curcumin was shown to effectively inhibit STAT3 and play anticancer roles in breast, ovarian, lung cancer, and esophageal squamous cell carcinoma.^399–401^ Moreover, the natural flavonoid myricetin was found to downregulate the levels of programmed death ligand-1 (PD-L1) and indoleamine 2, 3-dioxygenase 1 (IDO1) and restore T-cell activity and antitumor immunity by inhibiting the IFN-γ-activated JAK-STAT–IRF1 axis in lung A549, breast MDA-MB-231 and colon HCT116 cancer cells.^402,403^ Runtsch et al. proposed that the natural metabolite itaconate and its derivatives inhibit M2 polarization by blocking JAK1-STAT6 pathway phosphorylation, providing a novel perspective on M2 macrophage-driven diseases.^404^ As a new natural STAT3 inhibitor, XYA-2 was found to bind the SH2 domain of STAT3 and synergistically downregulate the levels of MYC and SLC39A10 in human gastric cancer cell lines and patient-derived xenograft (PDX) mouse models to perform its anticancer roles.^405^

In addition, SOCS proteins, as part of a negative feedback loop in the JAK-STAT pathway, and peptides targeting SOCS interactors have also shown promise as inhibitors of disease progression.^117,406,407^

Recently, several JAK inhibitors have been clinically approved for the treatment of various diseases, and a growing body of innovative drug candidates are in preclinical and clinical trials (Table 3). Here, we review the critical applications and adverse events of the major JAK inhibitors in human diseases.

**Table 3:** The applications and safety of main JAK inhibitors

JAK inhibitors	Selectivity	Approved indications	Indications under the clinical trials	Reported adverse events	Ref.
Tofacitinib	JAK1 and JAK3	rheumatoid arthritis, ulcerative colitis, juvenile idiopathic arthritis, and psoriatic arthritis	psoriasis, Crohn’s disease, COVID-19, alopecia areata, dermatomyositis, atopic dermatitis, keratoconjunctivitis sicca, relapsing polychondritis, ankylosing spondylitis, inflammatory bowel disease, and transplant rejection	infections, malignancies, anemia, neutropenia, elevated in serum creatinine and transaminases, hypercholesterolemia, gastrointestinal symptoms, and thromboembolism	^202,203,205,445–448,451–459,465–467^
Peficitinib	JAK3, JAK1, TYK2 and, JAK2	rheumatoid arthritis	ulcerative colitis and psoriasis	infections, malignancies, elevated creatine kinase, elevated creatinine, and hyperlipidemia	^185–188,192–194^
Ruxolitinib	JAK1 and JAK2	myelofibrosis and polycythaemia vera	psoriasis, polycythemia, vitiligo, malignancies, acute graft-versus-host disease, rheumatoid arthritis, essential thrombocythemia, alopecia areata, atopic dermatitis, COVID-19	anemia, thrombocytopenia, neutropenia, hypokalemia, infections, and peripheral edema	^206,411–417,419–425,437–442^
Baricitinib	JAK1 and JAK2	rheumatoid arthritis, atopic dermatitis, and COVID-19	lupus erythematosus, juvenile dermatomyositis, psoriasis, diabetic nephropathy, alopecia areata, and autoinflammatory diseases	infections, malignancies, and hyperlipidemia	^475,477–490^
Delgocitinib	JAK1, JAK2, TYK2, and JAK3	atopic dermatitis	eczema, discoid lupus erythematosus, psoriasis, and alopecia areata	nasopharyngitis, Kaposi’s varicella, contact dermatitis, and acne	^498–503^
Momelotinib	JAK1 and JAK2	/	myelofibrosis and multiple myeloma	diarrhea, cough, nausea, anemia, neutropenia, thrombocytopenia, and treatment-emergent peripheral neuropathy	^206–209,220–222^
Filgotinib	JAK1	rheumatoid arthritis	inflammatory bowel disease, psoriatic arthritis, and ankylosing spondylitis	infections, nasopharyngitis, and headache	^177–179,196–198^
Upadacitinib	JAK1	rheumatoid arthritis, ankylosing spondylitis, non-radiographic axial spondyloarthritis, and psoriatic arthritis	inflammatory bowel disease, and atopic dermatitis	infections, malignancies, elevated lipid parameters, increased creatine phosphokinase, increased hepatic aminotransferase, low blood cell counts, stroke, and venous thromboembolisms	^260–272,275–278^
Fedratinib	JAK2	myelofibrosis	thrombocytopenia and solid tumors	fatal encephalopathies, anemia, gastrointestinal symptoms, increased liver transaminases, increased serum creatinine, and increased pancreatic enzymes	^417,507–511^
Abrocitinib	JAK1	atopic dermatitis	psoriasis, systemic lupus erythematosus, and arthritis	nasopharyngitis, nausea, vomiting, acne, herpes zoster, increased blood creatine phosphokinase, dizziness, and headache	^283–287,289,528–531^
Decernotinib	JAK3	/	rheumatoid arthritis	headache, nausea, infections, elevated transaminases, lipoproteins, and creatinine, reduced lymphocyte and neutrophil	^513,515–517^
Itacitinib	JAK1	/	graft-versus-host disease and lung cancer	reduced platelet and neutrophil, anemia, and hyperglycemia	^518,519^
Deunavacitinib	TYK2	plaque psoriasis	psoriatic arthritis, systemic lupus erythematosus, and inflammatory bowel diseases	nasopharyngitis and upper respiratory tract infections	^534–537^

JAK inhibitors are small-molecule inhibitors that cause immunosuppression, reduce the pathological production of proinflammatory cytokines driven by JAK-STAT signaling, and inhibit gain-of-function JAK mutants.^408,409^ Various JAK inhibitors are currently in preclinical and clinical studies to treat a series of autoimmune diseases and cancers.^410–417^

The first-generation oral JAK inhibitors revolutionized the treatment of a group of heterogeneous disorders.^418^ The first clinically approved JAK inhibitor was the JAK1 and JAK2 inhibitor ruxolitinib, which was approved by the U.S. Food and Drug Administration (FDA) in 2011 to treat myelofibrosis.^206,419–425^ The COMFORT-I trial conducted by Verstovsek, S. et al. demonstrated the clinical benefits of ruxolitinib for patients with intermediate-2 or high-risk myelofibrosis; the ruxolitinib group showed improved manifestations, reduced spleen size, and elevated overall survival compared with the placebo group.^420^ Later, the COMFORT-II trial showed that patients with myelofibrosis benefited from continuous ruxolitinib therapy in comparison with patients that received the best available therapy, highlighting the long-term effectiveness and overall survival benefits of ruxolitinib.^426^ Kvasnicka, H. M. et al. reported that long-term administration of ruxolitinib for 48 months or 60 months ameliorated and stabilized the progression of bone marrow fibrosis.^427^ Zeiser et al. conducted a phase 3 trial to assess the efficacy of ruxolitinib in patients with glucocorticoid-refractory or -dependent chronic graft-versus-host disease. The results showed that 165 patients that received ruxolitinib manifested more favorable overall responses at 24 weeks, a longer duration of response at 12 months, and better failure-free survival than patients that received common second-line therapies.^428^ In a single-center retrospective study of patients with inflammatory bowel disease, 6 months of ruxolitinib treatment resulted in improved extraintestinal symptoms, stool frequency, steroid taper, and nutritional status.^429^ A case report suggested that the use of ruxolitinib early in the disease courses of refractory systemic idiopathic juvenile arthritis and interstitial lung diseases may block IFN-γ signaling to postpone disease-related deterioration.^430^ More recently, the RUXCOVID phase 3 trial randomly divided 432 patients into a ruxolitinib group (*n* = 287) and a placebo group (*n* = 145) plus standard of care to estimate the efficacy of ruxolitinib for the treatment of coronavirus disease 2019 (COVID-19). The results indicated that the median recovery time was 1 day less for patients treated with ruxolitinib compared with patients that received placebo.^431^ However, Fisher, D. et al. recently pointed out that multi-cytokine overproduction persisted in patients with myelofibrosis after treatment with ruxolitinib.^432^ Notably, a 75-year-old man with myelofibrosis was reported to develop progressive multifocal leukoencephalopathy after receiving ruxolitinib treatment.^433^ Several studies have also reported that the rapid discontinuation of ruxolitinib administration induced life-threatening withdrawal symptoms, which may be attributed to robust changes in the activities of inflammatory cytokines.^434–442^

The preferentially selective JAK3 and JAK1 inhibitor tofacitinib was the first JAK inhibitor approved for patients with RA that had poor responses to conventional drugs like methotrexate.^443,444^ Several studies showed that tofacitinib was consistently more efficacious and safer compared with other DMARDs in patients with RA.^445–448^ In recent years, tofacitinib exhibited promising efficacy in clinical trials for diverse autoimmune diseases.^203,449–459^ Tofacitinib was approved by the FDA for the treatment of active ulcerative colitis in 2018 and was also later validated in numerous studies to be safe and effective for the treatment of inflammatory bowel diseases.^460–467^ Sandborn, W. J. et al. performed three phase 3 trials and confirmed the favorable efficacy of tofacitinib as an induction and maintenance therapy in patients with moderately to severely active ulcerative colitis. Then, they further integrated several global clinical trials and validated the long-term safety and effectiveness of tofacitinib for patients with ulcerative colitis.^468^ In an open-label trial involving 10 patients with sarcoidosis, tofacitinib ameliorated skin and internal-organ symptoms in all 10 patients chiefly by suppressing type 1 immunity.^469^ You, H. et al. indicated that tofacitinib was the same or better than conventional immunosuppressants for improving the modified Rodnan skin score of patients with refractory skin thickening in diffuse cutaneous systemic sclerosis.^470^ Changelian, P. S. et al. demonstrated the potential benefit of tofacitinib for preventing allograft rejection in vivo by showing that tofacitinib mediated immunosuppression to prevent organ rejection in a murine model of heart transplantation and in a non-human primate model of kidney transplantation.^350^ To further explore the safety of tofacitinib in a real-world setting, Khosrow-Khavar, F. et al. enrolled two cohorts of patients with RA that received either tofacitinib or tumor necrosis factor (TNF) inhibitor and found a statistically insignificant increase in adverse cardiovascular outcomes in patients after tofacitinib treatment.^471^ However, the ORAL Surveillance reported that tofacitinib caused higher rates of infection and cardiovascular events compared with tumor necrosis factor inhibitors in patients with RA that were more than 50 years of age and had at least one additional cardiovascular risk factor. In preclinical trials and preclinical genetic studies, ruxolitinib was found to exhibit potential cardiotoxicity that may present as toxic myocardial damage, decreased left ventricular systolic function, or cardiac failure.^472–474^

The selective JAK1 and JAK2 inhibitor baricitinib was approved for the treatment of RA in 2017 and has been widely confirmed to improve clinical symptoms of RA in patients that have an inadequate response to methotrexate.^475,476^ The RA-BEAM trial involving patients with moderately to severely active RA and an inadequate response to methotrexate showed that baricitinib was superior to placebo with respect to signs and symptoms, physical function, and joint structural damage.^477–484^ Baricitinib also produced better improvements than adalimumab according to the criteria of the American College of Rheumatology (ACR20 response) and Disease Activity Score 28-joint count C reactive protein (DAS28-CRP).^485–490^ The RA-BEACON trials explored the efficacy of baricitinib in patients with RA that were refractory to bDMARDs. In two phase 3 trials in patients with severe alopecia areata, baricitinib was superior to placebo in producing hair regrowth.^487^. Baricitinib was also found to have a high affinity for numb-associated kinase (NAK) which enabled it to suppress NAK activity and block clathrin-mediated endocytosis to reduce viral infection in COVID-19.^491,492^ In addition, a pilot study showed that baricitinib improved respiratory symptoms and did not cause serious adverse events in 12 patients with mild-to moderate COVID-19 pneumonia.^493^ Cantini, F. et al. also performed a retrospective multicenter study of 113 consecutive patients hospitalized with moderate pneumonia and validated the beneficial effects of baricitinib in terms of reduced viral burden, mortality, and hospitalization rates.^494^ The ongoing RECOVERY trial further confirmed the effectiveness of baricitinib in reducing mortality due to COVID-19. An integrated study of 3,492 patients with RA that received baricitinib found no correlation between baricitinib use and major adverse cardiovascular events, arterial thrombotic events, or congestive heart failure.^495^

Peficitinib, a pan-JAK inhibitor that significantly inhibits JAK3, was approved for the treatment of RA in Japan in 2019.^496^ Numerous phase 2b (e.g., RAJ1, RA21, and RA22) trials have shown that peficitinib, as a monotherapy or combined with csDMARDs, achieved a rapid and statistically significant American College of Rheumatology (ACR) response rate over 12 weeks in patients with moderate-to-severe RA.^187,188,192^ The phase 3 trial (RAJ3 and RAJ4) also observed that peficitinib performs better in alleviating RA symptoms and joint destruction after 12 weeks of treatment. In addition, a phase 2b trial showed that patients with ulcerative colitis receiving ≥75 mg of peficitinib exhibited higher rates of clinical response, remission, and mucosal healing at week 8.^194^ Based on the outcomes of a phase 2a multicenter placebo-controlled study, peficitinib also exhibited dose-dependent improvements in clinical and histological lesions among patients with moderate-to-severe psoriasis.^193^ Furthermore, the safety profile of peficitinib is acceptable, commonly including nasopharyngitis, herpes zoster, diarrhea, increased blood creatine phosphokinase levels, and lymphopenia.^497^ Delgocitinib, a pan-JAK inhibitor with broad inhibitory effects on proinflammatory cytokines, is effective against diverse inflammatory skin conditions, including AD, eczema, discoid lupus erythematosus, psoriasis, and alopecia areata.^498–500^ In phase 3 trials, the topical administration of delgocitinib produced clinically meaningful improvements in efficacy endpoints for patients with moderate-to-severe AD, resulting in the approval of delgocitinib for AD treatment. Moreover, throughout the study periods in both children and adults with AD, the majority of adverse events were mild and not relevant to the use of delgocitinib.^501–504^

The first-generation JAK inhibitors are considered to be pan-JAK inhibitors that target multiple JAK isoforms. Given their highly conserved ATP-binding sites, JAK inhibitors produce a wide range of effects but also cause side effects including cardiovascular events, thromboembolic events, infection, and oncogenic complications.^505^ Therefore, the development of more selective JAK inhibitors with an emphasis on enhanced specificity and fewer adverse effects has become a focus of drug research.^506^ Fedratinib is a selective JAK2 inhibitor that was approved by the FDA in 2019 to treat patients with intermediate-2 or high-risk myelofibrosis.^507–511^ The National Comprehensive Care Network also classified fedratinib as a category 1 recommendation for higher-risk patients with myelofibrosis and platelet counts ≥50 × 10^9^/L.^512^ The randomized JAKARTA phase 3 trial and nonrandomized JAKARTA-2 phase 2 trial consistently showed that fedratinib attenuated symptoms and splenomegaly in patients with ruxolitinib-resistant or intolerant intermediate-1, intermediate-2, or high-risk myelofibrosis.^508^ In addition, Harrison, C. N. et al. conducted a phase 2 multicenter study and reported that treatment with 400 mg fedratinib resulted in remarkable reductions in spleen volume and clinical symptoms in ~30% of patients with myelofibrosis that was resistant or intolerant to ruxolitinib.^417^ However, fedratinib was reported to cause Wernicke’s encephalopathy, confirmed by magnetic resonance imaging, in 8 of 670 patients that received fedratinib treatment and in 4 women who received 500 mg/day fedratinib in the JAKARTA trial, which raised concerns and hampered the further clinical development of fedratinib.^510^ Anemia and thrombocytopenia were also observed in patients that received fedratinib treatment in both the JAKARTA trial and the JAKARTA-2 trial. Decernotinib, a JAK3-selective inhibitor, is currently under development and considered efficacious against RA.^513,514^ The previous phase 2a and 2b trials both reported that decernotinib, as a monotherapy or in combination with methotrexate, contributed to the improved signs and symptoms of RA patients with inadequate response to methotrexate.^515–517^ The major adverse effects seen in those trials included headache; nausea; elevated levels of transaminases, lipoproteins, and creatinine; and reduced numbers of lymphocytes and neutrophils.^516^ Itacitinib, a novel selective JAK1 inhibitor, has a wide range of anti-inflammatory activities. Thus far, itacitinib has been primarily used for the treatment of graft-versus-host disease (GVHD). A multicenter phase 2 trial (NCT03846479) recently reported that patients with low-risk acute GVHD administered 28 days of itacitinib without the conventional administration of systemic corticosteroids (SCSs) achieved effective drug response rates and reduced symptomatic flares.^518^ A phase 3 trial (GRAVITAS-301, NCT03139604) demonstrated that itacitinib, in combination with corticosteroids, did not attain the expected improvement in the overall response rate (ORR) at day 28 among patients with acute GVHD.^519^ The European Society for Blood and Bone Marrow Transplantation (EBMT 2022) reported the preliminary results of a phase 2 trial (NCT 04071366), stating that itacitinib exhibited good performance in the prevention of cytokine release syndrome (CRS) following by immunotherapies. Moreover, the World Congress of lung cancer (WCLC 2022) presented that patients with NSCLC with high PD-L1 expression (≥50%) had a favorable profile of efficacy and tolerance after 6 weeks of treatment with a combination of itacitinib and pembrolizumab. The most common adverse events mainly included decreased platelet and neutrophil counts, anemia, and hyperglycemia. Further studies are needed to obtain further insights into the use of itacitinib for the treatment of acute GVHD.

Currently, several JAK inhibitors in different phases of clinical trials are being explored to expand potential indications, increase drug adherence and achieve better safety. For example, upadacitinib (ABT‐494) is a potent and selective JAK1 inhibitor that received FDA approval for clinical use in patients with moderate-to-severe RA, psoriatic arthritis (PsA), ankylosing spondylitis (AS) and non-radiographic axial spondylarthritis (nr-axSpA).^520–522^ The SELECT-MONOTHERAPY study found that upadacitinib monotherapy improved the clinical symptoms when administered in combination with stable background csDMARDs in patients with moderate-to-severe active RA.^523^ Two classical phase 3 trials (SELECT-PsA 1 and SELECT-PsA 2) conducted in patients with PsA who had an inadequate or intolerant response to one or more TNF inhibitors found that upadacitinib reduced the PsA disease severity compared with placebo or adalimumab.^272,277^ Furthermore, upadacitinib was found to be effective in patients with active AS who had a bDMARD-inadequate response during a 14-week treatment period.^275^ In the SELECT-AXIS 2 trial, upadacitinib alleviated the painful symptoms of nr-axSpA as compared with placebo at week 14.^524^ Recently, the European Union passed approval for upadacitinib treatment to be developed for patients with inflammatory bowel disease based on positive clinical outcomes in two induction studies (U-ACHIEVE and U-ACCOMPLISH) and a maintenance study (U-ACHIEVE).^525^ Nonetheless, additional trials with large population samples are required to validate the long-term safety of upadacitinib, because other JAK inhibitors with similar mechanisms have reported adverse events such as cardiovascular events, embolism and thrombosis, cancer and even death.

Abrocitinib, another FDA-approved JAK1 selective inhibitor being developed for AD treatment, functions by blocking several critical cytokines, including IL-4, IL-13, IL-22, and IL-31, that participate in the pathology of AD.^526,527^ In a series of phase 3 trials (JADE Mono-1, MONO-2, TEEN, COMPARE, EXTEND, and REGIMEN), abrocitinib monotherapy achieved marked amelioration of pruritus and AD disease severity, resulting in the expansion of indications for abrocitinib administration to adolescents aged 12–17 years having moderate-to-severe AD.^284,285,287,528–530^ Furthermore, the rapid improvement of pruritus produced by abrocitinib supports treatment compliance, which further validates the safety and tolerability of abrocitinib in patients with moderate-to-severe AD.^531^ Abrocitinib has manageable safety, and its common adverse events include nausea, headache, acne and reduced platelet counts.^532,533^ Therefore, its benefit-to-harm ratio needs to be thoroughly investigated before it can be used for the long-term treatment of AD. Strikingly, deucravacitinib (BMS-986165), by producing a distinctively allosteric mechanism to combine with the TYK2 regulatory domain and not influence the functions of JAK1/2/3, became the first oral agent that was used to treat psoriasis with a favorable safety profile.^534^ Deucravacitinib is also the first approved TYK2 selective inhibitor for the treatment of moderate-to-severe plaque psoriasis; it was approved in September 2022 by the USA FDA and then approved in Japan for plaque, generalized pustular, and erythrodermic psoriasis.^535^ The two crucial phase 3 POETYK PSO-1 and POETYK PSO-2 trials have proven the superior performance of deucravacitinib against moderate-to-severe plaque psoriasis in terms of multiple efficacy endpoints compared with placebo or apremilast.^536,537^ Moreover, enduring symptom remission was still maintained at 52 weeks of continuous treatment with deucravacitinib. The most common adverse events of deucravacitinib include mild-to-moderate nasopharyngitis and upper respiratory tract infections. The recently announced POETYK PSO-3 trial broadens the exploration of the efficacy of deucravacitinib in Asian populations for the first time. The oral results of the 30th European Academy of Dermatology and Venereology (EADV) Congress demonstrated that the administration of deucravacitinib to patients with moderate-to-severe Asian plaque psoriasis results in sustained efficacy and even favorable efficacy for refractory scalp psoriasis. Meanwhile, the efficacy and safety of deucravacitinib in a variety of immune-related diseases, including psoriatic arthritis (PsA), systemic lupus erythematosus, and inflammatory bowel disease, is also under exploration in multiple large clinical trials.^538^

In recent years, several independently developed Chinese JAK inhibitors (e.g., including jaktinib, golidocitinib, ivarmactinib, and itacitinib) under different phases of clinical trials are being considered to have great clinical potential.^539–543^ In October 2022, Jaktinib, a deuterated compound of momelotinib that widely inhibits JAK1, JAK2, JAK3, TYK2 and ACVR1, became the first approved domestic JAK inhibitor for the treatment of moderate-to-severe myelofibrosis.^544,545^ A published phase 2 trial has shown that jaktinib reduced the spleen size and alleviated the clinical symptoms of patients with moderate-to-severe myelofibrosis.^546^ It was evident that jaktinib was well-tolerated and that anemia and thrombocytopenia were the most common adverse events. The phase 3 trial (NCT04617028) further reported the encouraging efficacy of jaktinib in the treatment of myelofibrosis, with remarkable improvement of the symptoms, anemia and spleen volume when compared with hydroxyurea. Based on the reported existing efficacy and safety, approvals were needed for conducting clinical trials on the use of jaktinib in a series of diseases, such as COVID-19, AD, systemic lupus erythematosus, alopecia areata, and mild-to-moderate psoriasis.^547^

Overall, JAK inhibitors offer great promise as targeted therapies. However, sufficient real-world evidence is required to fully explore their long-term safety, durability and effectiveness.

## Current challenges and directions in the field

A sophisticated understanding of JAK-STAT signaling has provided many insights into the development of novel drugs.^548–550^ However, many challenges remain for improving target selectivity in JAK-STAT signaling, and exploration of the long-term safety of the developed agents is still required. Each JAK inhibitor impedes the combination of a JAK enzyme with ATP through the highly selective ATP-binding pocket.^551,552^ The Michaelis equilibrium of ATP, JAK enzyme and JAK inhibitor shows that the combination of JAK with its substrate is affected by the intracellular drug concentration and drug selectivity.^553^ However, drug concentrations depend on multiple factors, such as the patient’s age, body weight, liver and renal function and drug interactions, and it has been challenging to achieve absolute selectivity in terms of JAK inhibitors.^140,554^ More selective JAK inhibitors are being developed to reduce their unwanted effects on cytokine functions and further improve the overall safety and efficacy.^555–557^ For example, the JAK inhibitors abrocitinib and delgocitinib are currently being further developed to optimize treatment efficiency with minimum off-target effects.

Combination therapies using JAK inhibitors combined with other immunomodulatory or anti-inflammatory agents are an emerging approach; these are also being explored to achieve maximum treatment response with few adverse events.^558,559^ The simultaneous inhibition of the JAK-STAT signaling pathway and other potential targets appears to be another feasible option for drug development. For example, fedratinib, an innovative JAK inhibitor that simultaneously targets the activities of JAK2 and Fms-like tyrosine kinase 3 (FLT3) with crucial effects on the survival and proliferation of primitive hematopoietic progenitor cells, has been approved as an oral treatment for intermediate or high-risk myelofibrosis.^560–562^

The topical or inhalational application of JAK inhibitors is another exciting area of research in multiple ongoing animal models and larger clinical trials.^548,563^ The intestinally restricted pan-JAK inhibitor TD-1473 is expected to soon become available to treat inflammatory bowel disease with fewer systemic adverse side effects than other treatment options.^564^ The ongoing development and approval of emerging JAK inhibitors in preclinical studies and clinical settings will provide optimized JAK inhibitors as potent therapeutic options for a range of diseases in the future.

## Conclusions

As a primarily canonical signaling pathway, the JAK-STAT signaling pathway transduces cytokine-activated extracellular signals to the nucleus to mediate gene expression, thus exerting indispensable functions in a series of cellular processes, particularly ones with immunomodulatory effects. Abnormal activation of JAK-STAT signaling is central to the initiation and progression of many diseases, especially immune-related diseases, and cancers. Drugs that target the JAK-STAT pathway have been approved by the FDA as alternative treatments for certain diseases and have exhibited powerful clinical benefits. A substantial number of novel agents targeting the JAK-STAT pathway are currently under development, yet there is still little evidence of differences in efficacy between selective JAK inhibitors and pan-selective JAK inhibitors. Explorations of the use of JAK inhibitors in early disease stages and in combination with other conventional drugs are also currently in progress. Recent studies have shown that a portion of patients are unresponsive to JAK inhibitors and have tried to unveil the relevant drug resistance mechanisms. Future research should offer comprehensive insights into the physiological and pathogenic mechanisms of the JAK-STAT pathway in disease development and aim to identify biomarkers to assess the long-term efficacy and safety of JAK inhibitors and to optimize the therapeutic efficacy of JAK inhibitors in patients with different stages and severity of disease to achieve individualized treatment. Overall, understanding the associations of the JAK-STAT pathway with immune regulation and disease progression will provide new therapeutic strategies to treat diverse diseases, particularly immune-related diseases, and cancer.
